# Fermentation Optimization of Ergosta‐4,6,8(14),22‐Tetraen‐3‐One From *Aspergillus oryzae* and Its Anti‐Inflammatory Mechanism via Multi‐Pathway Inhibition of MyD88/NF‐κB/MAPK/NLRP3 Signaling

**DOI:** 10.1002/fsn3.71347

**Published:** 2025-12-15

**Authors:** Bingye Yang, Shining Liu, Yu‐Wei Chang, Chao Yi, Minxin You, Yung‐Husan Chen

**Affiliations:** ^1^ Xiamen Key Laboratory of Natural Products Resources of Marine Medicine Xiamen Medical College Xiamen China; ^2^ Fujian Provincial University Marine Biomedical Resources Engineering Research Center Xiamen Medical College Xiamen China; ^3^ Department of Food Science National Taiwan Ocean University Keelung Taiwan

**Keywords:** anti‐inflammatory, ergosta, fermentation, optimization

## Abstract

*Aspergillus oryzae* is a key microorganism in the production of various traditional food products, including rice wine, soy sauce, and baijiu. In this study, we identified and quantified ergosta‐4,6,8 (14), 22‐tetraen‐3‐one (ETO), a bioactive compound derived from *A. oryzae* , using high‐performance liquid chromatography (HPLC). Our results confirmed that ETO is one of the major components produced by *A. oryzae* . Bioactivity assays of the crude extract revealed that ETO exhibits significant anti‐inflammatory properties. To enhance ETO production, we optimized the fermentation medium. Single‐factor experiments identified glycerol and N‐acetylglucosamine as the optimal carbon and nitrogen sources, respectively. An orthogonal experimental design was then employed to further refine the fermentation conditions, resulting in the determination of an optimal medium composition. The feasibility and effectiveness of this optimized fermentation process were validated, laying the groundwork for the industrial‐scale production of ETO. To elucidate the anti‐inflammatory mechanism underlying ETO's bioactivity, further investigations were performed in LPS‐stimulated RAW264.7 macrophages. The results demonstrated that ETO significantly reduced the secretion of pro‐inflammatory cytokines IL‐6, IL‐1β, and TNF‐α, and suppressed the activation of the MyD88/NF‐κB/MAPK/NLRP3 signaling pathway, indicating its potential as a potent anti‐inflammatory agent. In conclusion, ETO is not only abundantly produced by *A. oryzae* , but also exhibits pronounced anti‐inflammatory activity, providing a promising foundation for its future development in anti‐inflammatory therapeutics.

## Graphical abstract

This study identified ergosta‐4,6,8(14),22‐tetraen‐3‐one (ETO) as a major anti‐inflammatory compound in *Aspergillus oryzae* and optimized fermentation conditions (glycerol and N‐acetylglucosamine as carbon/nitrogen sources) to enhance ETO yield. ETO suppressed IL‐6, IL‐1β, and TNF‐α in LPS‐stimulated macrophages by inhibiting the MyD88/NF‐κB/MAPK/NLRP3 pathway, demonstrating its therapeutic potential. The findings provide a foundation for scaling ETO production and advancing its application in anti‐inflammatory therapies.

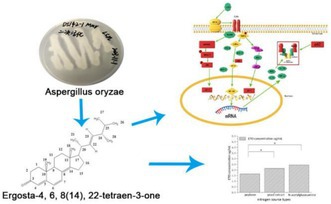

## 1 Introduction

ETO belongs to the steroidal compounds and serves as a precursor for synthesizing vitamin D. It is a conjugated ketone with an isoprene molecular structure, with a chemical formula of C_28_H_40_O and a relative molecular weight of 392. It exists as yellow or pale yellow crystals (Chen et al. 2014). ETO exhibits absorption at 360 nm in long wavelengths and shows bright yellow spots upon charring with sulfuric acid, enabling its simple identification via thin‐layer chromatography (TLC). These substances also constitute the main components of fungal cell membranes, participating in various complex reactions within organisms. By binding with phospholipids, they stabilize membrane structure, maintaining the stability and permeability of fungal cell membranes. Furthermore, research has unveiled numerous pharmacological and physiological activities associated with ETO (Huang et al. 2021). Within the intricate structure of the human body, ETO not only binds with phospholipids in cell membranes but also combines with lipopolysaccharides (LPS) to suppress the production of inflammatory cytokines, including interleukin (IL)‐1β, IL‐6, and IL‐8. Studies indicate that ergosterol binding with lipids A can alleviate LPS‐induced inflammation, thus preventing its induction (Choi et al. 2021). Additionally, research results demonstrate the anti‐inflammatory activity of ETO against LPS‐induced macrophages (Nowak et al. 2022).

*Aspergillus oryzae* is commonly found in nature, particularly in grains and fermented foods, and serves as a traditional microbial strain in the production of soy sauce, alcoholic beverages, and other fermented products in China. For instance, during soy sauce production, the proteases produced by *A. oryzae* not only break down raw materials to determine the content of amino acid nitrogen but also release amino acids or peptides by degrading proteins, thereby enhancing the flavor, color, and taste of soy sauce (Fan et al. 2021). Additionally, Yuan et al. (2020) leveraged the amylase‐producing capability of *A. oryzae* and co‐fermented it with yeast to produce a type of rice wine characterized by its natural and pure aroma, along with a mellow taste. In recent years, Qiao et al. (2010) isolated ETO from *A. oryzae* using a potato‐glucose‐based fermentation medium. Therefore, these findings demonstrate that under specific conditions, *A. oryzae* can convert nutrients into ETO through its unique metabolic pathways. Compared with other production methods, microbial fermentation for ETO production offers several advantages, including a shorter production cycle, stable yield, and a fermentation broth and mycelium rich in nutrients. Furthermore, this method is less affected by external factors, easier to control (Zhao et al. 2020), Presently, various medicinal fungi have been found to contain ETO, such as medicinal gastrointestinal fungi (such as coarse Lasiosphaera seu Calvatia, purple bald Lasiosphaera seu Calvatia, etc.), *Polyporus umbellatus*, Boletinus pictus, Fomitiporia ellipsoidea, etc. (Xu et al. 2019; Guo et al. 2019; Du et al. 2012; Song et al. 2015), all of which have been found to contain ETO. ETO, being a primary component of various medicinal fungal cell membranes, possesses diuretic properties and is used in treating chronic kidney diseases and as an anti‐tumor agent (Guo et al. 2023). In this study, a steroid compound named ETO was isolated from the ethyl acetate extract of *A. oryzae* . *A. oryzae* as a key flavor‐fermenting microorganism in soy sauce, contained significant amounts of ETO, playing a crucial role in its flavor development (Qiao et al. 2010). Though microbial fermentation for producing ergosterol has been reported, its low content in fermentation products limits its application in relevant fields (Lou et al. 2020). Therefore, large‐scale fermentation of ETO by *A. oryzae* holding significant research value was also investigaged in this study. Meanwhile, due to its effectiveness in LPS induced mouse monocyte macrophage RAW264.7, the anti‐inflammatory mechanism of ETO was explored from the perspectives of inflammatory factors and signaling pathways.

## 2 Materials and Methods

### 2.1 Main Instruments and Reagents

RAW264.7 murine macrophage cell line (CL‐0190, Wuhan Pricella Biotechnology Co. Ltd.), PM150210 DMEM high glucose culture medium (Wuhan Punuosi Life Science and Technology Co. Ltd.); fetal bovine serum (FND500, Ecocyte Bioscience Co. Ltd.); LPS (L4130, Sigma Corporation); CCK8 assay kit (BS380A, Biyuntian Biotechnology Co. Ltd.); nitric oxide assay kit (S0021S, Biyuntian Biotechnology Co. Ltd.); ELISA assay kit (EMC001b and EMC004, Xinbosheng Biotechnology Co. Ltd.); reverse transcription kit (RR047A, TaKaRa Corporation); BCA assay kit (B3101050, Yisheng Biotechnology Co. Ltd.); RIPA cell lysis buffer (R0020, Solabo Technology Co. Ltd.); ND2000 (21806498, Quawell Corporation); Cycle i160 cell culture incubator, ABI7500 real‐time fluorescence quantitative PCR instrument (Thermo Fisher Scientific); high‐speed refrigerated centrifuge (5810R, Eppendorf Corporation).

#### 2.1.1 Reagents and Materials

The reagents used in this study included potato dextrose agar and broth culture media from Guangdong Huanke Microbial Technology Co. Ltd.; yeast extract powder, protein peptone, soluble starch, sucrose, glucose, glycerol, ethyl acetate, methanol, and acetic acid from Xilong Technology Co. Ltd.; methanol (chromatographically pure) from Guangzhou Tongyuan Chemical Technology Co. Ltd.; and an ETO standard sample from Xiamen Medical College, Department of Marine Pharmacy Teaching and Research Group.

The instruments and equipment included a fully automatic high‐pressure sterilizer (GR85DR) from Zhiwei Instrument Co. Ltd.; a clean bench (SW‐CJ‐2FD) from Sujing Antai; a biochemical incubator (SPX‐250B‐Z) from Shanghai Boxun Industry Co. Ltd.; a light shaking bed (ZAZY‐CGF8) from Shanghai Zhichu Instrument Co. Ltd.; an electric constant temperature blast drying oven (DHG‐9140A/*) from Shanghai Ailang Instrument Co. Ltd.; an ultrasonic cleaning machine (DS‐080S) from Dongsen; a rotary evaporator (Re212‐B) from Yamato; a low‐temperature coolant circulation pump (DLSB‐10/20) from Zhengzhou Changcheng Science and Technology Trade Co. Ltd.; an electronic balance (BSA224S) from Saitoris Scientific Instrument (Beijing) Co. Ltd.; a high‐performance liquid chromatography instrument (1260 Infinity) from Agilent Technologies; a column chromatography cylinder from Supelco Corporation; and a solid‐phase extraction column from Nanochrom.

#### 2.1.2 Strain and Fermentation Conditions

The Strain A.oryzae, purchased from Guangdong Microbial Culture Collection Center (registration number GDMCC61933). The seed culture media include:

*Solid medium*: Potato dextrose agar powder culture medium (PDA), initial salinity suitable for 15 ppt seawater, initial pH value 7.2–7.4 (adjusted with 3 mol/L HCl and 6 mol/L NaOH), sterilized in an autoclave at 121°C for 2 h.

*Seed liquid culture medium*: Potato dextrose broth powder culture medium (PDB), initial salinity suitable for 15 ppt seawater, initial pH value 7.2–7.4 (adjusted with 3 mol/L HCl and 6 mol/L NaOH), sterilized in an autoclave at 121°C for 2 h.

*Fermentation culture medium*: Carbon source single‐factor fermentation culture medium, nitrogen source single‐factor fermentation culture medium, orthogonal optimization fermentation culture medium, initial salinity suitable for 15 ppt seawater, initial pH value 7.2–7.4 (adjusted with 3 mol/L HCl and 6 mol/L NaOH), sterilized in an autoclave at 121°C for 2 h (Figure S2).

### 2.2 Strain Activation

The refrigerated strain A.oryzae was transferred from the −80°C refrigerator to the −20°C refrigerator for 20 min, then transferred to the −4°C refrigerator for 20 min, and finally placed at room temperature for 20 min. The bacterial solution was coated on PDA medium and cultured at 25°C for 3–5 days.

### 2.3 Subculture of Strain

From the successfully activated strain medium, single colonies were extracted by inoculation ring and transferred to the new PDA medium by plate scribing method for further culture at 25°C for 3–5 days.

### 2.4 Extended Culture of Strain

200 μL PDB medium was transferred to the successful strain medium and repeatedly blown, and then sucked back into PDB medium to make seed liquid, which was cultured in shaker incubator for 48–72 h (rotation speed 150 r/min).

After the seed solution was prepared, 5 mL seed solution was absorbed and evenly spread into the rice medium (100 g rice, 250 mL ultrapure water), and the culture was sealed at room temperature for 2 months.

### 2.5 Organic Extraction

Pour 200 mL of mixed solvent (ethyl acetate: methanol: acetic acid = 80:15:5) into rice culture medium and extract with ultrasound for 30 min. Then pour out the extract and repeat this step 4–5 times. Afterwards, all extracts were concentrated by rotary evaporation to obtain 146.73 g of extract.

### 2.6 Silica Gel Column Chromatography

The established method was used for silica gel column separation (Chen et al. 2024). DS142‐1‐2‐1 (1.2312 g) was obtained by separating DS142‐1‐2 (2.3215 g) through SephadexLH‐20 gel column (mobile phase was dichloromethane and methanol = 1:1), and three components were obtained, DS142‐1‐2‐1 (1.2312 g), DS142‐1‐2‐2 (490.05 mg), and DS142‐1‐2‐3 (598.11 mg). DS142‐1‐2‐1 components were superimposed on a normal atmospheric column to obtain 3 components. DS142‐1‐2‐1‐2 (583 mg) was obtained by thin layer chromatography (TLC) to determine the Rf value of the compound and the solvent and ratio of the developing agent (*n*‐hexane: acetone = 4:1), and then TLC was prepared by silica gel plate to obtain 2 components.

After TLC preparation, the sample DS142‐1‐2‐1‐2‐1 (67.3 mg) was analyzed in liquid phase and the liquid phase preparation conditions were determined (Welch Ultimate XB‐C18 column, 5 μm, 10 × 250 mm, monitored at 210 and 254 nm, flow rate: 2 mL/min with 100% methanol) to obtain pure compound ergosta‐4, 6, 8 (14), 22‐tetraen‐3‐one (ETO) (15.8 mg).

### 2.7 Single‐Factor Impact Experiment

The established method was employed to evaluate the impact of different carbon and nitrogen sources on ETO production (Chen et al. 2024). Effect of carbon‐to‐nitrogen ratio on ETO Production: Based on the optimal carbon source (glycerol) and nitrogen source (N‐acetylglucosamine), experiments were conducted to assess the influence of varying carbon‐to‐nitrogen (C/N) ratios. Fermentation media with C/N ratios of 1:7, 1:8, and 1:9 were tested, with each condition replicated three times. After fermentation, ETO in the metabolic products was qualitatively and quantitatively analyzed using HPLC. The optimal C/N ratio for ETO production was determined by integrating HPLC results, standard curve analysis, and statistical processing via SPSS software (Figure S3).

### 2.8 Orthogonal Design Optimization of Fermentation Medium

Building upon the optimal carbon source (glycerol) and nitrogen source (N‐acetylglucosamine) identified in the single‐factor experiments, design L9 (3^2^) orthogonal array to optimize the composition of *A. oryzae* fermentation medium. Orthogonal design and corresponding factor levels are shown in Table 1.

**TABLE 1:** Levels of factors in the orthogonal experiment.

Factor	Levels		
	−1	0	1
Glycerol (g/L)	35	40	45

### 2.9 Fermentation Culture and Sample Treatment and Determination of ETO Content

The established method was used for the experiment (Chen et al. 2024). Construct a standard curve (Figure S4).

### 2.10 Statistical Analysis Methods and Data Processing

Statistical analyses were conducted using GraphPad Prism 8.0.2. Data normality was assessed via the Shapiro–Wilk test, with *p* > 0.05 indicating a normal distribution. One‐way analysis of variance (ANOVA) was used for intergroup comparisons, while single‐sample *t*‐tests were performed to calculate mean and standard deviation. Results were presented as mean ± standard deviation (SD), with statistical significance set at *p* < 0.05. ETO concentrations were calculated based on peak areas from HPLC results. The mean concentrations of parallel experiments for each experimental factor level were calculated using Microsoft Excel software. To compare significant differences between different levels of the same influencing factor, IBM SPSS Statistics 27 software was used. Graph plotting was conducted using Origin 2022 software.

### 2.11 Detection of the Toxic Effect of ETO on RAW264.7 Cells

For in vitro evaluation, murine monocyte‐derived macrophages in the logarithmic growth phase were seeded into 96‐well plates at a density of 8 × 10^4^ cells per well in 200 μL of suspension and incubated at 37°C with 5% CO_2_ until fully adherent. Experimental groups were as follows: (1) Normal control (cells + complete medium), (2) Blank control (complete medium only), and (3) Treatment groups (cells + complete medium + ETO at graded concentrations). ETO was prepared in basic medium at final concentrations of 1, 10, 20, 30, 40, and 50 μg/mL, with five replicates per condition. After 24 h, cell viability was evaluated using the CCK‐8 assay to determine the optimal ETO concentration.

### 2.12 Detection of the Anti‐Inflammatory Effect of ETO on LPS‐Induced RAW264.7 Cells

RAW264.7 cells were assigned to three groups: (1) Normal control (cells + complete medium), (2) LPS group (cells + complete medium +100 ng/mL LPS), and (3) Experimental group (cells + complete medium + 100 ng/mL LPS + varying concentrations of ETO). The cell inoculation density was a density of 8 × 10^4^ cells per well in 200 μL of suspension, each concentration was tested in five replicates. After 4 h of culture, LPS (100 ng/mL) was added to both the LPS and experimental groups. Following 24 h of incubation at 37°C with 5% CO_2_, nitric oxide (NO) levels in the supernatant were quantified using a nitric oxide assay kit to determine the optimal ETO concentration.

### 2.13 Detection of the Effect of ETO on Inflammatory Factors in LPS‐Induced RAW264.7 Cells

RAW264.7 cells were divided into three groups: (1) Normal control (cells + complete medium), (2) LPS group (cells + complete medium + LPS), and (3) Experimental group (cells + complete medium + LPS + ETO at 30 or 40 μg/mL), with five replicates per concentration. Cells were seeded into 6‐well plates and cultured as described in Section 2.8. Following NO quantification to assess the anti‐inflammatory effects of ETO, supernatants were collected for further analysis of IL‐6 and IL‐1 by our laboratory.

### 2.14 Detection of the Effect of ETO on the Expression of Inflammatory Factor mRNA in LPS‐Induced RAW264.7 Cells

Experimental procedures were conducted as described in Section 2.9. After collecting cell supernatants, total RNA (1 μg) was extracted from cell pellets using Trizol reagent. cDNA was synthesized with a reverse transcription kit, and inflammatory factor mRNA expression was quantified via RT‐qPCR using an ABI 7500 system, with β‐actin as the internal reference gene. Primer sequences and qPCR conditions are provided in Table 2.

**TABLE 2:** Primers and operating conditions for qPCR.

Primer name	Primer sequence
MCP‐1	Forward: GCTGTAGTTTTTGTCACCAAGC Reverse: GTGCTGAAGACCTTAGGGCA
IFN‐β	Forward: GGAAAGATTGACGTGGGAGA Reverse: CTGAGGCATCAACTGACAGG
INOS	Forward: ACTCAAGAATGGTCGCGAGG Reverse: GTGCCATCAGAGCAGTCTGT
IL‐1β	Forward: ATGGCAACTGTTCCTGAACTC Reverse: GCCCATACTTTAGGAAGACA
TNF‐α	Forward: CGGTGCCTATGTCTCAGCCT Reverse: GAGGGTCTGGGCCATAGAAC
IL‐6	Forward: AGTTGCCTTCTTGGGACTGA Reverse: TCCACGATTTCCCAGAGAAC
MIP‐2	Forward: CCAGACAGAAGTCATAGCCACT Reverse: GGTTCTTCCGTTGAGGGACA
β‐Actin	Forward: TCATCACTATTGGCAACGAGC Reverse: AACAGTCCGCCTAGAAGCAC

### 2.15 Effect of ETO on the Expression of Key Signaling Pathway Proteins in LPS‐Induced RAW264.7 Cells

Cells were divided into three groups: (1) Normal control (cells + complete medium), (2) LPS group (cells + complete medium + LPS), and (3) Experimental group (cells + complete medium + LPS + ETO at 30 or 40 μg/mL), with three replicates per concentration. Experimental procedures followed those in Section 2.10. After collecting cell pellets, proteins were extracted, and the expression levels of p‐Erk1/2, Erk1/2, MyD88, NF‐κB, NLRP3, p62, p‐IκB, IκB, JNK, and p‐JNK were analyzed by Western blotting.

## 3 Results

**FIGURE 1:**
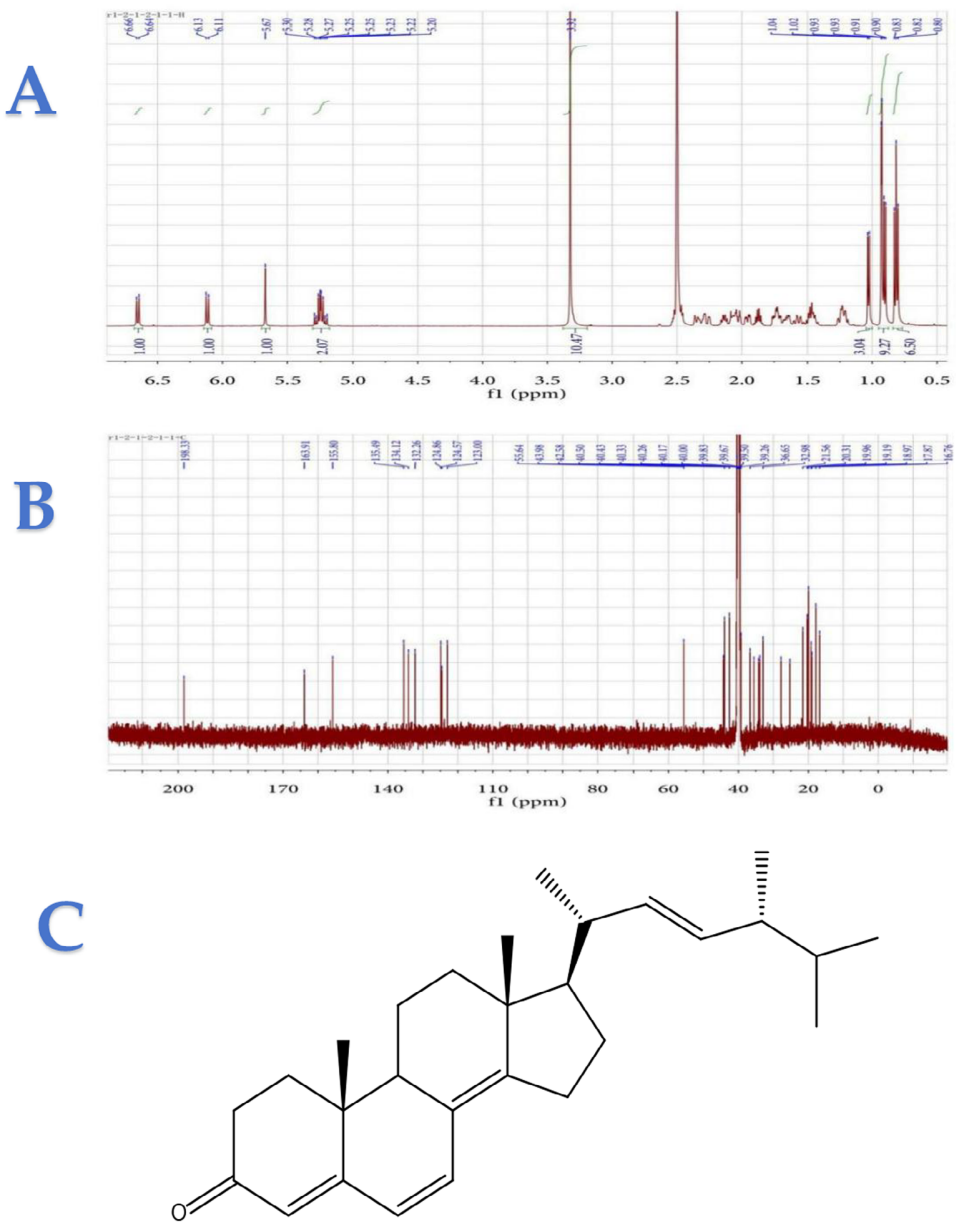
NMR spectra of ETO (500 MHz, DMSO‐d_6_): (A) ^1^H NMR spectrum, (B) ^13^C NMR spectrum, and (C) the structure of ETO.

In this study, steroid compounds, specifically ETO, a yellow oil, were isolated and purified from the secondary metabolites of the fungus *Aspergillus oryzae*. The structural elucidation of ETO (as shown in Figure 1) was conducted through analysis of its ^1^H‐NMR and ^13^C‐NMR spectra (as shown in Figure 1) and by comparison with previously reported data in the literature (Gao et al. 2001; Qiao et al. 2017). Furthermore, the anti‐inflammatory activity of ETO was evaluated using lipopolysaccharide‐induced RAW 264.7 cells, demonstrating its potential therapeutic effects.

### 3.1 HPLC Analysis of Standard ETO and Crude Extract

**FIGURE 2:**
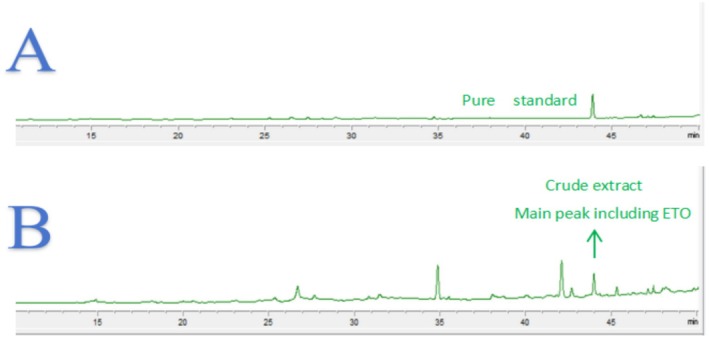
HPLC profiles of ETO and the crude extract (A: HPLC profile of ETO, B: HPLC profile of the crude extract from *A. oryzae* ).

Figure 2 shows the HPLC profiles of the standard ETO (A) and the crude extract obtained from *A. oryzae* (B). The analysis indicates that ETO is one of the major components in the crude extract. Subsequent bioactivity assays demonstrated that ETO exhibits significant anti‐inflammatory activity. However, the limited yield of ETO from the initial fermentation process posed a challenge for conducting detailed mechanistic studies. To address this issue and ensure sufficient quantities of ETO for in‐depth investigations into its anti‐inflammatory mechanisms, fermentation optimization was undertaken. This approach not only enhanced the production efficiency of ETO but also provided a more robust platform for exploring its potential as a therapeutic agent.

### 3.2 Single‐Factor Experimental Results

#### 3.2.1 Effect of Carbon Source Types on ETO Production

**FIGURE 3:**
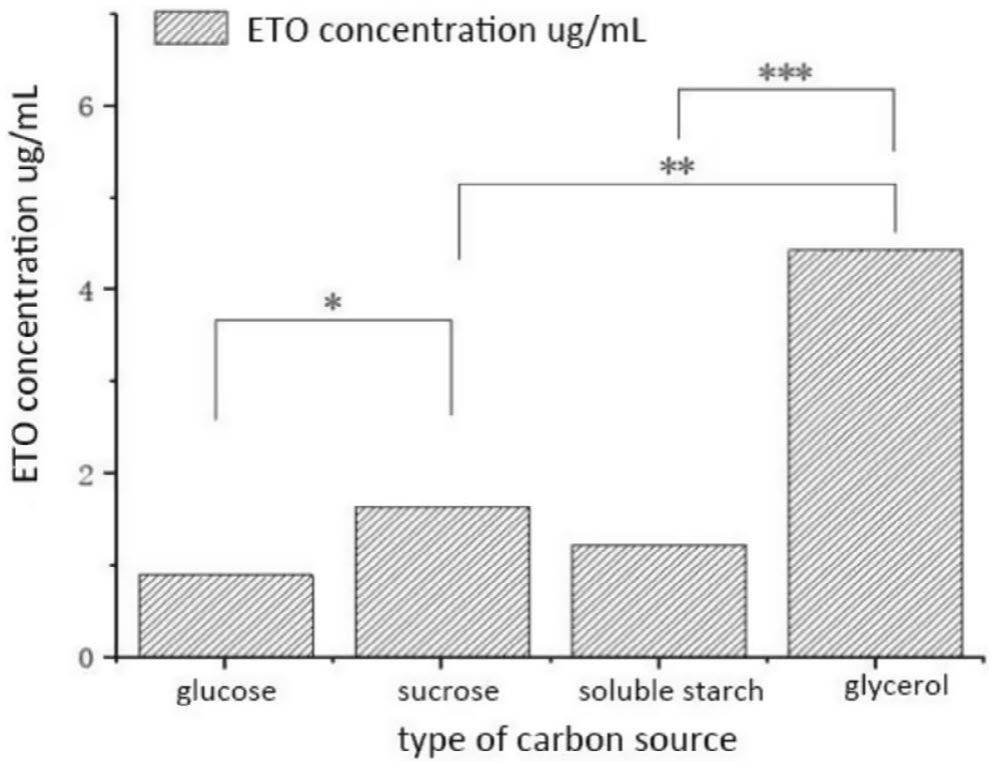
Effect of different carbon sources on ETO production by *A. oryzae* . (*p < 0.05; **p < 0.01; ***p < 0.001).

Carbon sources are essential for microbial metabolism, supplying energy and carbon skeletons for secondary metabolites. Figure 3 (Dunnett T3, *p* < 0.05) shows that glycerol yielded the highest ETO production (4.43 μg/mL) in *A. oryzae* , followed by sucrose, while soluble starch and glucose had minimal impact, likely due to enzyme system differences. Thus, glycerol was chosen as the optimal carbon source.

#### 3.2.2 Effect of Nitrogen Source Types and Carbon‐Nitrogen Ratio in the Culture Medium on ETO Production

**FIGURE 4:**
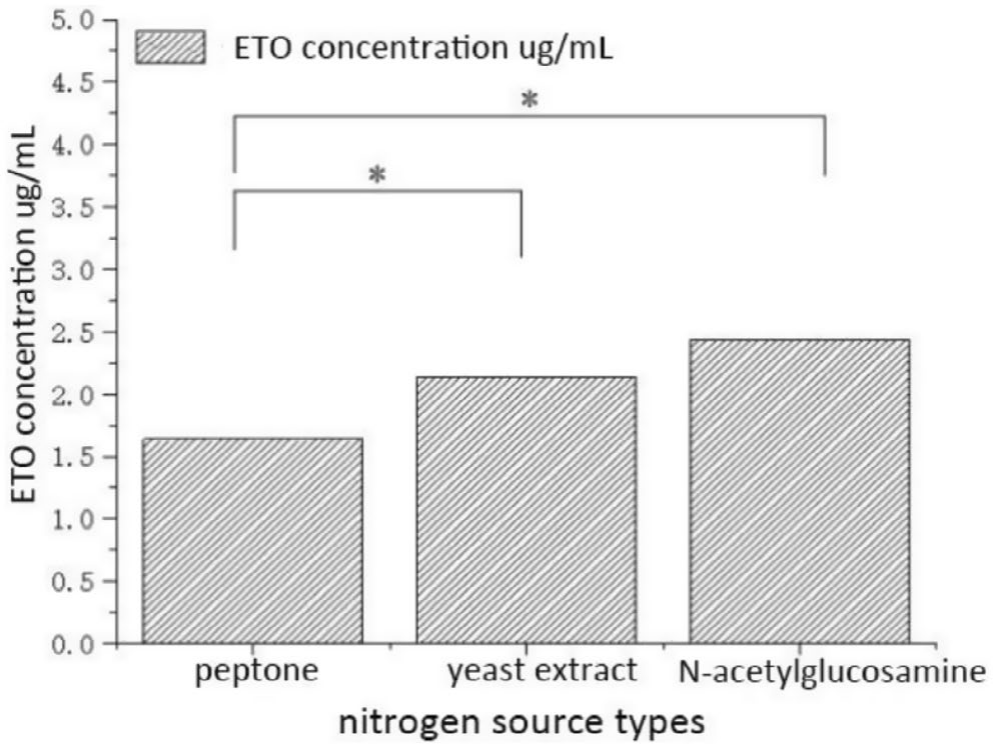
Effect of different nitrogen sources on ETO production by A.oryzae. *p < 0.05 (significant difference within the control group).

Nitrogen sources are essential nutrients for microbial growth and metabolism (Chen, Bian, et al. 2018; Chen, Conos, et al. 2018). As shown in Figure 4 (analyzed using the Dunnett T3 method, *p* < 0.05), a single‐factor experiment assessing different nitrogen sources revealed that N‐acetylglucosamine yielded the highest ETO production (2.44 μg/mL), followed by yeast extract, while peptone resulted in the lowest yield. Thus, N‐acetylglucosamine was selected as the optimal nitrogen source. Additionally, the effect of the C/N ratio on ETO production was investigated. Statistically significant differences were observed at C/N ratios of 1:7, 1:8, and 1:9, with the highest ETO yield (0.27 μg/mL) occurring at a C/N ratio of 1:8 (Figure S1).

### 3.3 Results and Analysis of Orthogonal Experimental Design

Based on the single‐factor results of each influencing factor and its levels, glycerol concentration and N‐acetylglucosamine concentration in the culture medium were selected as variables, using the L9 (3^2^) orthogonal table. The L9 (3^2^) orthogonal experimental table and experimental results processing are shown in Table 3, homogeneity test of variance in Table 4 and analysis of variance data processing in Table 5.

**TABLE 3:** SPSS designed the table and its experimental results.

Number	A (glycerol concentration)	B (N‐Acetylglucosamine concentration)	Ceramide concentration of 1 (μg/mL)	Ceramide concentration 2 (μg/mL)	Ceramide concentration 3 (μg/mL)
1	1	1	0.36	0.37	0.36
2	1	2	2.29	2.14	2.06
3	1	3	3.65	3.72	3.74
4	2	1	0.30	0.26	0.29
5	2	2	0.25	0.25	0.26
6	2	3	0.25	0.18	0.21
7	3	1	0.27	0.29	0.28
8	3	2	0.68	0.65	0.66
9	3	3	0.10	0.10	0.11
K1	18.69	2.78			
K2	2.25	9.24			
K3	3.14	12.06			
K1/9	2.08	0.31			
K2/9	0.25	1.03			
K3/9	0.35	1.34			
R	1.83	1.03			

**TABLE 4:** Table of homogeneity of variance tests.

*F*	df 1	df 2	Conspicuousness
684.132	8	18	*p* < 0.001

**TABLE 5:** Analysis of variance table.

Source of variance	Type III sum of squares	df	Mean square	*f*‐number	Conspicuousness
A	18.995	2	9.498	4757.590	*p* < 0.001
B	5.030	2	2.515	1259.763	*p* < 0.001
A × B (interactive)	12.237	4	3.059	1532.417	*p* < 0.001
Deviation	0.036	18	0.002		
Total of corrected	36.297	26			

Analysis in Table 3, it can be intuitively analyzed that the optimal combination for ETO production by *A. oryzae* is A1B3. This involves using glycerol as the carbon source with a concentration of 35 g/L, and using N‐acetylglucosamine as the nitrogen source with a concentration of 20 g/L.

Table 4 indicates significant differences between them when glycerol is used as the carbon source and when N‐acetylglucosamine is used as the nitrogen source. There is also a notable difference in the interaction effect between glycerol and N‐acetylglucosamine. To validate this orthogonal result, *A. oryzae* was cultured under optimal conditions, and the ETO content was measured. The result closely matched the data in the orthogonal table, confirming the feasibility of this experimental design.

### 3.4 Toxicity Assessment of ETO on Mouse RAW264.7 Cells

**FIGURE 5:**
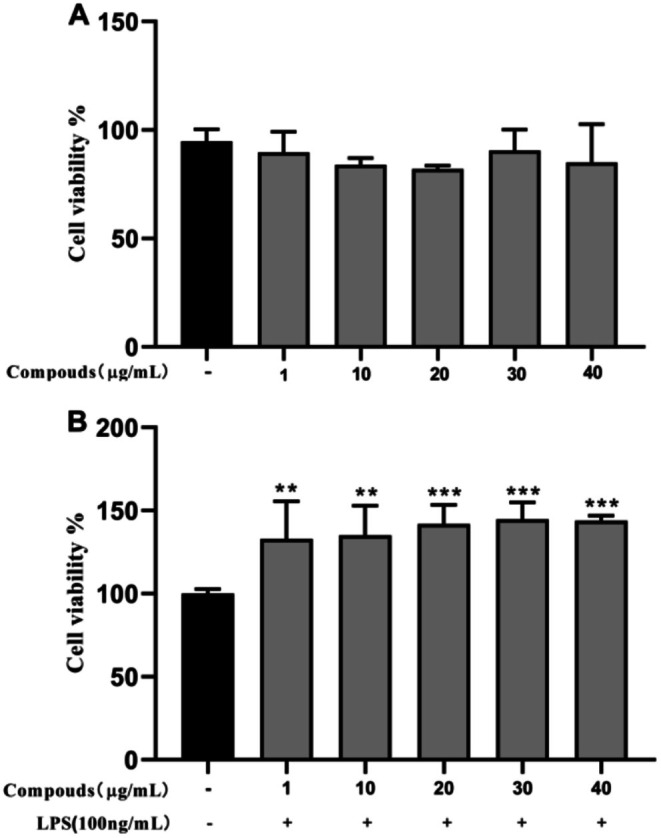
The toxic effect of ETO and ETO with LPS on RAW264.7 cells. Data are expressed as mean ± SD from five independent experiments. **p < 0.01, ***p < 0.001 (vs. normal control).

**FIGURE 6:**
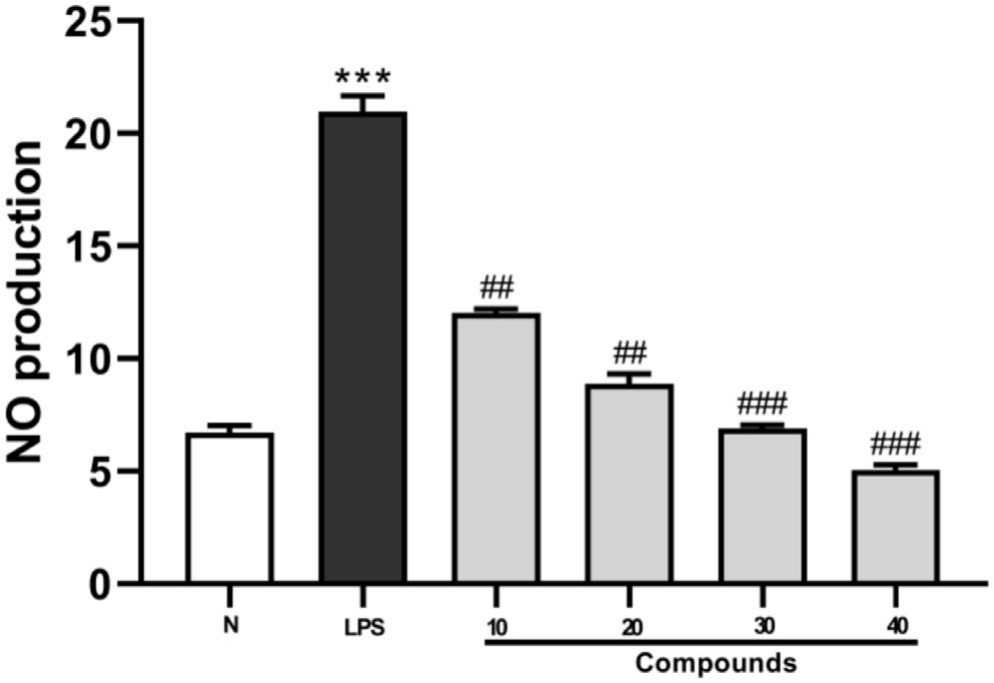
The anti‐inflammatory effects of ETO on LPS‐induced RAW264.7 cells were evaluated. Data are expressed as mean ± SD from five independent experiments. ***p < 0.001 (vs. normal control); ##*p* < 0.01, ###*p* < 0.001 (vs. LPS‐treated group).

As shown in Figure 6A, compared to the control group, the cell viability values in the experimental groups with different concentrations of ETO were slightly increased, but no significant differences were observed. This indicates that ETO had no toxicity in mouse RAW264.7 cells within this experimental concentration range. As shown in Figure 5B, compared to the control group, the ETO + LPS co‐treatment group exhibited a significant increase in cell viability, suggesting enhanced cellular proliferation. This observation further confirms the absence of cytotoxicity in RAW264.7 cells under ETO + LPS co‐treatment conditions.

### 3.5 Anti‐Inflammatory Effect of ETO on LPS‐Induced Mouse RAW264.7 Cells

Figure 6 illustrates a significant increase in nitric oxide (NO) levels in the LPS‐stimulated group compared to the control (*p* < 0.001), confirming the successful induction of inflammation. In contrast, NO production was substantially reduced in the groups treated with different concentrations of ETO, underscoring its anti‐inflammatory effects against LPS‐induced macrophage activation (*p* < 0.01). ETO at concentrations of 30 and 40 μg/mL demonstrated the strongest anti‐inflammatory responses (*p* < 0.001). Consequently, these concentrations were selected for further anti‐inflammatory evaluations in 6‐well plates.

### 3.6 Detection Results of ETO on the mRNA Expression of IL‐6, IL‐1β, TNF‐α, IFN‐β, MCP‐1, MIP‐2, and INOS in LPS‐Induced Mouse Monocyte Macrophages

**FIGURE 7:**
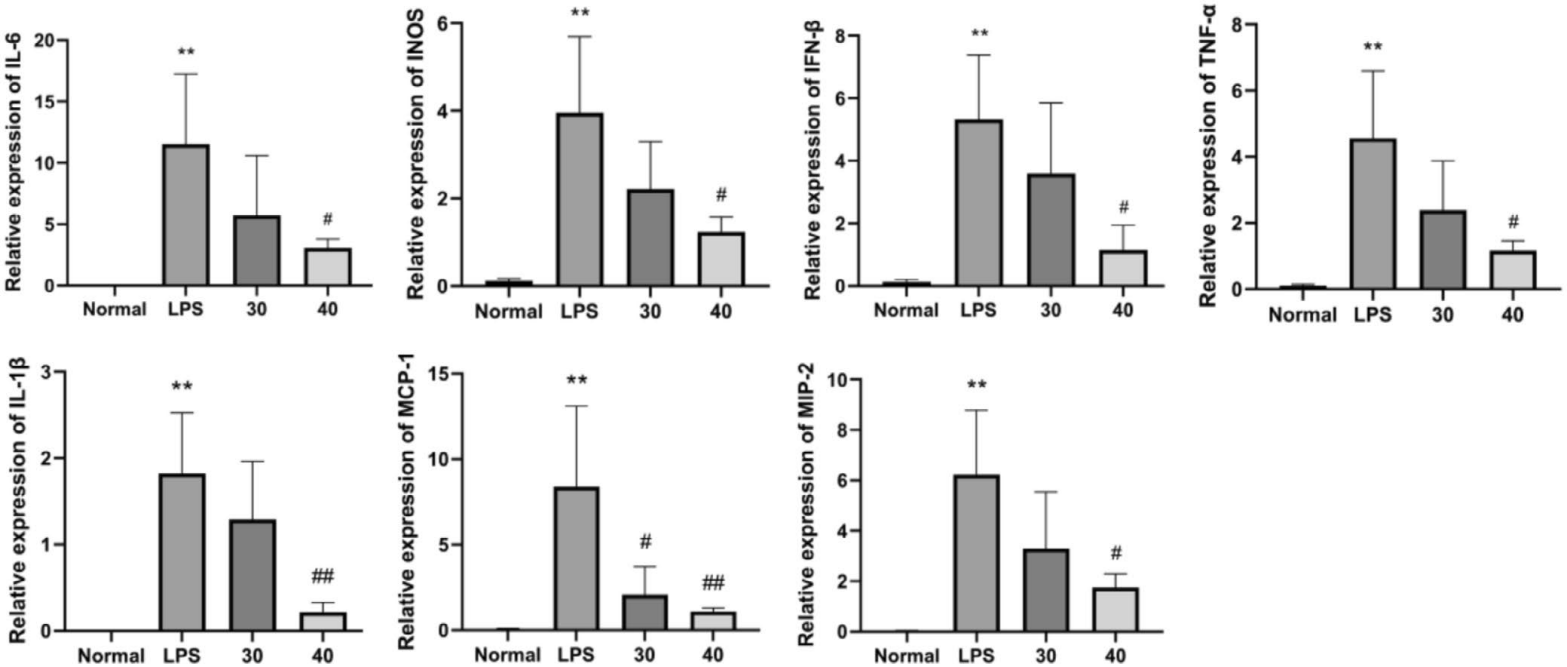
The protein levels of IL‐6, IL‐1β, TNF‐α, MCP‐1, MIP‐2, and iNOS in RAW264.7 cells stimulated with LPS were assessed following ETO treatment. Data are presented as mean ± SD from five independent experiments. Statistical analysis revealed significant differences: **p < 0.0001 (normal group vs. LPS‐treated group) and ^#^ *p* < 0.05; ^##^ *p* < 0.01 (LPS‐treated group vs. ETO‐treated group).

Figure 7 demonstrates a marked increase in inflammatory markers in the LPS‐exposed group compared to the control group (*p* < 0.01). This confirmed the successful induction of inflammation. Compared to the LPS group, the 30 μg/mL ETO treatment showed no significant alterations in pro‐inflammatory mediator expression, except for a pronounced reduction in MCP‐1 levels. However, at 40 μg/mL, ETO markedly suppressed the expression of IL‐6, IL‐1β, TNF‐α, and other inflammatory markers. In the ETO‐treated group (40 μg/mL), mRNA expression of IL‐6, IL‐1β, TNF‐α, IFN‐β, MCP‐1, MIP‐2, and iNOS was significantly downregulated relative to the LPS group (*p* < 0.05). These results indicate that ETO effectively inhibits the upregulation of IL‐6 and other pro‐inflammatory mediators in LPS‐activated RAW264.7 macrophages.

### 3.7 Detection Results of ETO on LPS‐Induced Mouse RAW264.7 Cell p‐Erk1/2, Erk1/2, MyD88, NF‐κB, NLRP3, p62, p‐IκB, IκB, p‐JNK, and JNK Protein Expression

**FIGURE 8:**
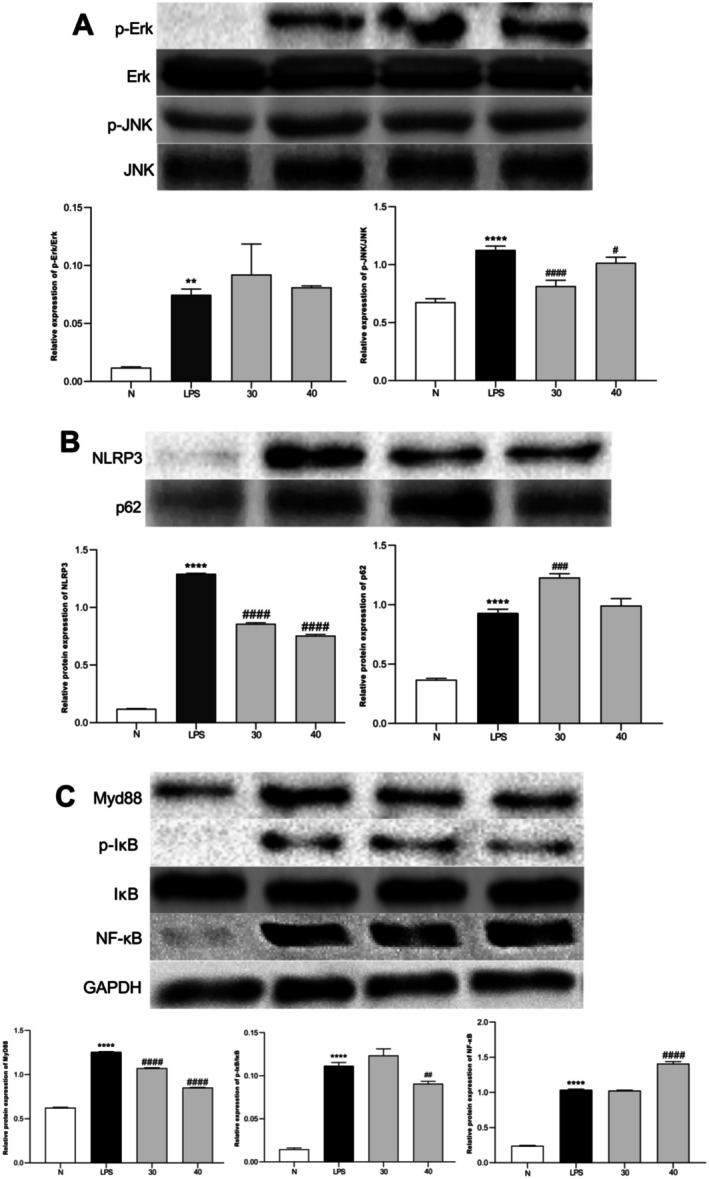
ETO exerts its anti‐inflammatory effects by modulating the MAPK, NF‐κB, and NLRP3 signaling pathways. (A) Protein expression of p‐Erk1/2, Erk1/2, p‐JNK, JNK, and GAPDH in the MAPK pathway; (B) Protein expression of NLRP3, p62, and GAPDH in the NLRP3 pathway; (C) Protein expression of MyD88, NF‐κB, p‐IκB, IκB, and GAPDH in the NF‐κB pathway. Data are presented as mean ± SD from three independent experiments. Statistical significance: *p < 0.0001 (vs. normal control); ####*p* < 0.0001 (vs. LPS‐treated group). The internal reference protein GAPDH is suitable for calibrating all other quantitative proteins.

Figure 8A reveals marked activation of the MAPK pathway in the LPS‐treated group, with elevated levels of phosphorylated Erk1/2 (p‐Erk1/2) and phosphorylated JNK (p‐JNK) compared to the total protein levels of Erk1/2 and JNK. the control. In the ETO‐treated group, p‐JNK levels were significantly reduced, while p‐Erk1/2 expression remained unchanged. These findings indicate that ETO suppresses the MAPK/JNK signaling pathway, thereby attenuating inflammatory responses. Analysis of the NLRP3 inflammasome pathway revealed a marked upregulation of NLRP3 protein expression in LPS‐stimulated macrophages compared to the control group, whereas ETO treatment significantly reduced NLRP3 levels. Notably, LPS exposure increased p62 protein expression relative to the control, while ETO administration restored p62 expression to levels exceeding those observed in the LPS group.

Regarding the NF‐κB pathway, LPS treatment led to significant upregulation of MyD88, NF‐κB, and phosphorylated IκB (p‐IκB) compared to the control. ETO treatment notably decreased MyD88 and p‐IκB expression, while NF‐κB levels remained unaffected. These findings suggest that ETO attenuates inflammatory responses by suppressing the MyD88/IκB signaling pathway.

## 4 Discussion

The optimal carbon and nitrogen sources were identified through single‐factor impact experiments. Glycerol was determined as the optimal carbon source, while N‐acetylglucosamine was identified as the optimal nitrogen source. Based on the single‐factor results, glycerPrevious studies have established the presence of ergosta‐4,6,8(14),22‐tetraen‐3‐one (ETO) in the fermentation systems of *Marasmius epiphyllus* strain, with solid‐state fermentation yielding an average content of 4.8 μg/g (Lou et al. 2020). In contrast, liquid fermentation in our study demonstrated a slightly lower ETO concentration (3.7 μg/mL). Despite this quantitative difference, liquid fermentation offers distinct advantages for industrial‐scale production, including scalability and process control. ETO has garnered significant attention in clinical drug development due to its confirmed pharmacological activities. Research indicates that ETO exerts therapeutic effects by modulating serum creatinine (SCr) and blood urea nitrogen (BUN) levels, while concurrently inhibiting renal interstitial fibrosis through anti‐fibrotic mechanisms (Zhao et al. 2011). Supported by these pharmacological properties, ETO has been granted a national patent in China, underscoring its application in medications targeting chronic renal failure. To further advance its industrial potential, this study systematically optimized the fermentation medium for ETO production by *Aspergillus oryzae*, focusing on carbon and nitrogen source selection. Glycerol concentration and N‐acetylglucosamine concentration were selected as the two influencing factors for orthogonal research, each with three suitable levels. The optimal fermentation combination was determined to be A1B3. Validation of the optimized fermentation conditions for *A. oryzae* revealed that 35 g/L glycerol and 8 g/L N‐acetylglucosamine yielded an ETO content of 3.70 μg/mL. This study highlights that microbial metabolite production during fermentation depends on both medium composition and fermentation conditions. Further research is required to establish an experimental and theoretical foundation for industrial‐scale ETO production through microbial fermentation.

The optimal carbon and nitrogen sources for ETO production were identified through single‐factor experiments, with glycerol and N‐acetylglucosamine selected as the most effective substrates. An orthogonal design (L9 (3^2^)) was subsequently employed to optimize their concentrations, revealing that 35 g/L glycerol and 20 g/L N‐acetylglucosamine yielded the highest ETO content (3.70 μg/mL). Although this concentration is slightly lower than that obtained via solid‐state fermentation in *Marasmius epiphyllus* (4.8 μg/g), liquid fermentation offers superior industrial scalability, environmental control, and reproducibility. This systematic optimization not only improves ETO yield but also establishes a foundation for large‐scale microbial production.

Importantly, while ETO is a defined pure compound with well‐characterized pharmacological activities—particularly anti‐inflammatory and anti‐fibrotic effects—the crude ethyl acetate extract of *Aspergillus oryzae* showed even stronger bioactivity in preliminary assays (data not shown). This discrepancy may be attributed to synergistic interactions among co‐extracted metabolites, which could enhance cellular uptake, prolong half‐life, or modulate multiple signaling pathways simultaneously. Therefore, future studies should investigate the comparative pharmacodynamics of the extract and purified ETO. These include potential additive or synergistic effects and the contribution of other fungal sterols or secondary metabolites. Understanding these differences will not only guide formulation strategies but also maximize the therapeutic potential of fermentation‐derived fungal products.

LPS, a structural component of the Gram‐negative bacterial cell wall, interacts with the TLR4 receptor on macrophage membranes upon host infection, triggering intracellular signaling cascades. These cascades activate various pathways, including NF‐κB and MAPK, and elicit pro‐inflammatory effects (Miyake 2004; Chen, Bian, et al. 2018; Chen, Conos, et al. 2018; Hoffmann and Baltimore 2006). Upon TLR4 activation, the inflammatory response is transmitted through two primary pathways: NF‐κB and MAPK (Akira and Takeda 2004). The NF‐κB pathway utilizes MyD88 as an adaptor protein, with TLR4's intracellular TIR domain binding to MyD88's carboxy‐terminal. This MyD88‐IL‐1R‐associated protein complex promotes TRAF6 oligomerization, activating the downstream NF‐κB signaling pathway (O'Neill and Bowie 2007; McGettrick and O'Neill 2004). NF‐κB is a heterodimer composed of p65 and p50 subunits, inhibited in the cytoplasm by IκB. Autophosphorylation of IκB triggers its degradation, releasing and activating NF‐κB molecules (Zhong et al. 1997).

In this study, compared with the normal control group, LPS stimulation significantly increased the protein expression levels of MyD88, p‐IκB, and NF‐κB, indicating NF‐κB pathway activation. In the ETO‐treated group, MyD88 and p‐IκB levels were significantly reduced, suggesting that ETO inhibits MyD88 expression, decreases IκB phosphorylation, and prevents its ubiquitination and degradation. Consequently, NF‐κB activation is reduced, leading to an anti‐inflammatory effect. The NF‐κB pathway regulates inflammatory factor expression, with studies showing its activation promotes IL‐1β, IL‐6, and TNF‐α synthesis (Udenigwe et al. 2013; Zhou et al. 2008). Consistent with this, our results showed increased mRNA expression of IL‐1β, IL‐6, and TNF‐α in the LPS group, while the ETO group exhibited significant reductions. This suggests that ETO inhibits NF‐κB pathway activation, reducing inflammatory cytokine production. Additionally, MIP‐2, which enhances TLR4 expression and activates macrophages, plays a key role in late‐stage inflammation and cytokine storm formation (Jin et al. 1999; Liu et al. 2020). Our results showed that ETO significantly reduced MIP‐2 mRNA expression, further supporting its NF‐κB inhibitory effect.

The MAPK pathway, a classical inflammatory signaling cascade, regulates cellular processes such as proliferation, apoptosis, and differentiation (Arthu and Ley 2013; Kim and Choi 2010). TLR4 activation induces TRAF6 oligomerization, which activates ECSIT or TAK to phosphorylate MEKK1 or MKK6, respectively. These pathways activate downstream MAPK components ERK, p38, and JNK (Kyriakis and Avrush 2001). Their phosphorylation promotes TNF‐α, IL‐1β, and IL‐6 synthesis (Yang et al. 2014). Moreover, inhibiting ERK suppresses iNOS and arginase II expression in LPS‐stimulated macrophages, achieving anti‐inflammatory effects (Cui et al. 2021).

Our findings revealed that LPS significantly increased p‐Erk1/2 protein expression, indicating MAPK/ERK pathway activation. However, ETO treatment did not significantly affect p‐Erk1/2 levels, suggesting ETO does not target this pathway for anti‐inflammatory action. Conversely, p‐JNK protein expression was significantly reduced in the ETO group, indicating inhibition of the MAPK/JNK pathway. The JNK signaling pathway, activated by LPS, phosphorylates and promotes inflammatory cytokine release, including TNF‐α and IL‐1β. ETO's inhibition of p‐JNK suggests a mechanism for its anti‐inflammatory activity. Additionally, MCP‐1 expression, regulated by MAPK pathways and ion channel activity, was significantly reduced in the ETO group, further corroborating ETO's inhibitory effect on MAPK/JNK‐mediated inflammation (Zhao 2013).

The NLRP3 inflammasome consists of the adaptor protein ASC, Caspase‐1, and NLRP3 protein, which generate mature IL‐1β and participate in adaptive immunity (Kanneganti et al. 2007). IL‐1β secretion and NLRP3 expression are key signals for inflammasome activation (Chen et al. 2014). In this study, ETO significantly reduced IL‐1β and NLRP3 protein expression, indicating inhibition of inflammasome assembly. p62, a selective autophagy adaptor protein, binds ubiquitinated ASC molecules, facilitating NLRP3 inflammasome degradation via autophagy (Zhang et al. 2021). Compared to the normal group, LPS stimulation significantly increased p62 expression. However, ETO treatment further increased p62 levels at 30 μg/mL, suggesting that ETO promotes autophagy‐mediated NLRP3 degradation, independent of p62 protein changes, thereby reducing inflammation.

**FIGURE 9:**
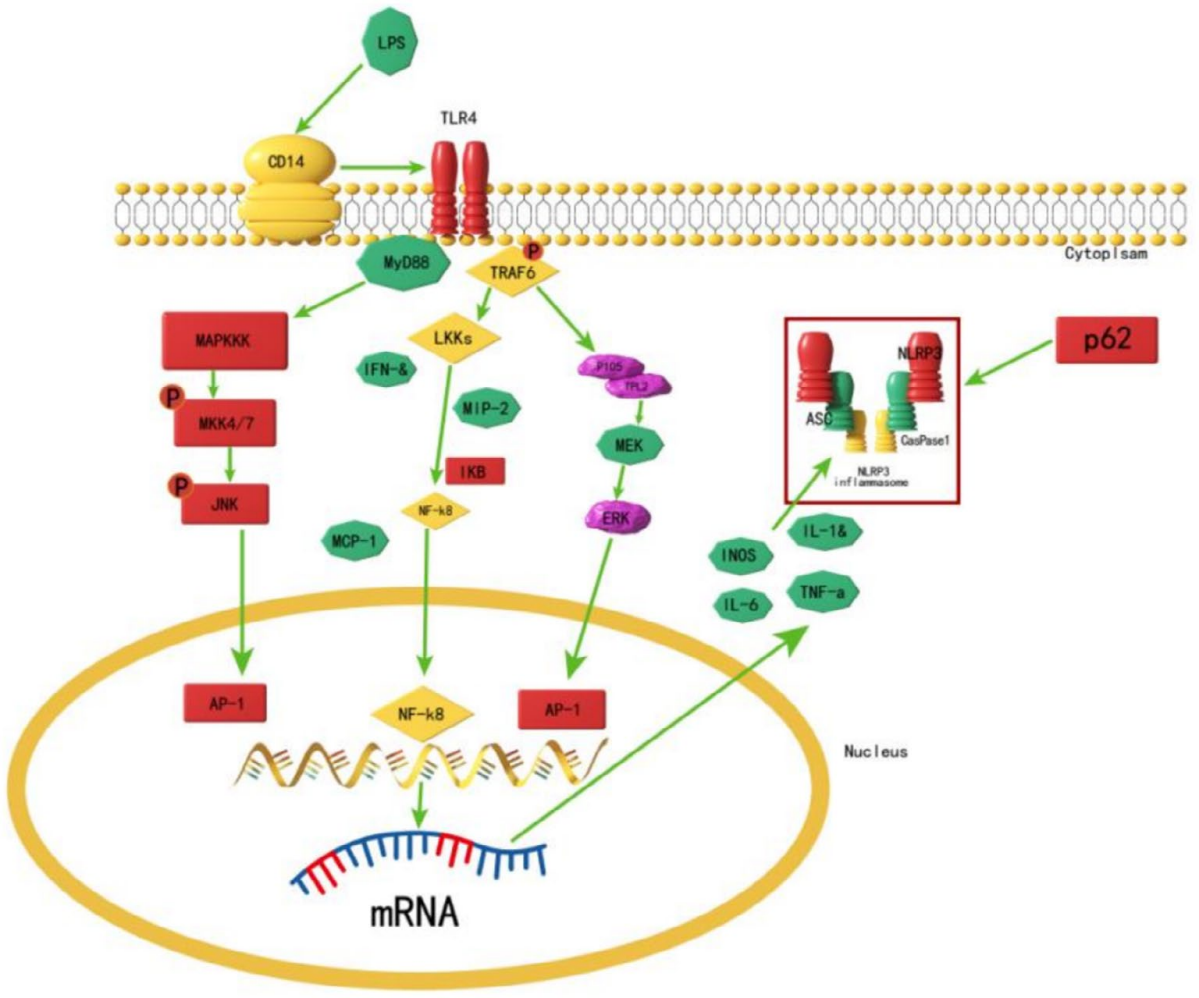
Anti‐inflammatory mechanism of ETO in LPS‐induced RAW264.7 macrophages.

In summary, as depicted in Figure 9, ETO reduces MyD88 protein expression, inhibiting the NF‐κB and MAPK/JNK pathways. This suppression decreases transcription, production, and release of inflammatory factors, including IL‐6, IL‐1β, TNF‐α, MCP‐1, MIP‐2, and iNOS. Furthermore, ETO inhibits NLRP3 expression and promotes p62‐mediated inflammasome degradation, ultimately mitigating the inflammatory response and exerting anti‐inflammatory effects.

## Author Contributions

**Bingye Yang:** conceptualization (equal), funding acquisition (equal), investigation (equal), project administration (equal), resources (equal). **Shining Liu:** data curation (equal), formal analysis (equal), investigation (equal), methodology (equal), software (equal), writing – original draft (lead). **Yu‐Wei Chang:** writing – review and editing (supporting). **Chao Yi:** data curation (equal), formal analysis (equal), methodology (equal). **Minxin You:** writing – review and editing (lead). **Yung‐Husan Chen:** conceptualization (equal), funding acquisition (equal), resources (equal), supervision (lead), visualization (lead).

## Funding

This research was funded by the following sources: Technology Innovation Program of Xiamen Ocean and Fisheries Development Special Funds, grant no. 23YYZP004QCB28. Science and Technology Program of Fujian Province, grant no. 2023J011659. Joint Funds for the innovation of Science and Technology, Fujian Province, grant no. 2024Y9717. Natural Science Foundation of Fujian Province, grant no. 2023J011651. Xiamen Municipal Directive Healthcare Program (3502Z20214ZD1325).

## Disclosure

Patents: All authors have reviewed and approved the final manuscript. Contributor roles are detailed according to the CRediT taxonomy. Authorship is limited to individuals who have made significant contributions to the research presented.

## Conflicts of Interest

The authors declare no conflicts of interest.

## Supporting information


**Figure S1:** Effect of different carbon‐to‐nitrogen ratios in culture medium on ETO production by A.oryzae.
**Figure S2:** Fungal culture on PDA medium.
**Figure S3:** ETO standard curve.
**Figure S4:** The stimulating effects of different concentrations of LPS.

## Data Availability

All data supporting the findings of this study are included in the manuscript and its Supporting Information.

## References

[fsn371347-bib-0001] Akira, S. , and K. Takeda . 2004. “Toll‐Like Receptor Signalling.” Nature Reviews Immunology 4, no. 7: 499–511. 10.1038/nri1391 15229469

[fsn371347-bib-0002] Arthu, J. S. C. , and S. C. Ley . 2013. “Mitogen‐Activated Protein Kinases in Innate Immunity.” Nature Reviews Immunology 9, no. 13: 679–692. 10.1038/nri3495 23954936

[fsn371347-bib-0003] Chen, H. , D. Q. Chen , Q. F. Li , P. F. Li , H. Chen , and Y. Y. Zhao . 2014. “Research on Pharmacological Activity, Pharmacokinetics and Content Determination of Ergosterone.” Journal of Traditional Chinese Medicine 39, no. 20: 3905.

[fsn371347-bib-0004] Chen, L. C. , S. A. Conos , B. Unal , and V. Tergaonkar . 2018. “Noncoding RNAs: Master Regulators of Inflammatory Signaling.” Trends in Molecular Medicine 24, no. 1: 66–84. 29246760 10.1016/j.molmed.2017.11.003

[fsn371347-bib-0005] Chen, Q. Q. , C. H. Bian , Y. B. Kang , S. M. Zhao , and S. J. Li . 2018. “Isolation and Identification of an Agricultural Antagonistic Actinomycete Strain.” Chinese Tobacco Science 24, no. 6: 134–139.

[fsn371347-bib-0006] Chen, Y.‐H. , Q. Q. Zhu , J. Y. Li , et al. 2024. “Optimization of Fermentation Process for New Anti‐Inflammatory Glycosylceramide Metabolite From *Aspergillus* sp.” Metabolites 14, no. 2: 99. 38392991 10.3390/metabo14020099 PMC10890386

[fsn371347-bib-0007] Choi, J.‐H. , H.‐J. Lee , S.‐E. Park , S. Kim , K.‐S. Seo , and K.‐M. Kim . 2021. “Cytotoxicity, Metabolic Enzyme Inhibitory, and Anti‐Inflammatory Effect of Lentinula Edodes Fermented Using Probiotic Lactobacteria.” Journal of Food Biochemistry 45: e13838. 10.1111/jfbc.13838 34212412

[fsn371347-bib-0008] Cui, X. , Z. F. Zheng , Y. Li , X. H. Jia , Y. Wang , and Q. Q. Yao . 2021. “Research Progress on the Biological Activity of Podopsin.” Chinese Journal of Modern Applied Pharmacy 38, no. 9: 1133–1139.

[fsn371347-bib-0009] Du, Z. W. , J. K. Liu , C. Xiang , and G. Wang . 2012. “Chemical Composition Study of Tiger Skin Calf Liver Bacteria.” Natural Product Research 24, no. 5: 618–621.

[fsn371347-bib-0010] Fan, J. X. , S. Liu , X. Y. Lu , and J. Chen . 2021. “Breeding of *Aspergillus* Rice Strain and Its Influence on Flavor Generation of Soy Sauce.” Food and Fermentation Industries 47, no. 21: 1–8.

[fsn371347-bib-0011] Gao, J. M. , L. Hu , and J. K. Liu . 2001. “A Novel Sterol From Chinese Truffles Tuber Indicum.” Steroids 66, no. 10: 771–775. 11522340 10.1016/s0039-128x(01)00105-2

[fsn371347-bib-0012] Guo, G. G. , J. M. Yun , B. Wang , Y. X. Zhao , W. H. Li , and Y. L. Qu . 2023. “Effect of Six Chinese Herbal Extracts on Ergosterol in Submerged Fermentation of *Morchella esculen* .” Food and Fermentation Industries 49, no. 20: 38–44.

[fsn371347-bib-0013] Guo, N. , Z. Y. Wu , J. G. Wang , Y. S. Sun , and H. Wang . 2019. “Analysis of the Content of Three Effective Components in Poria Cocos From Different Origins, Growth Years, and Harvesting Periods.” Special Wild Economic Animal and Plant Research 41, no. 1: 72–77 +94.

[fsn371347-bib-0014] Hoffmann, A. , and D. Baltimore . 2006. “Circuitry of Nuclear Factor KappaB Signaling.” Immunological Reviews 210: 171–186. 16623771 10.1111/j.0105-2896.2006.00375.x

[fsn371347-bib-0015] Huang, H. , L. Wang , Z. H. Hu , and B. Zeng . 2021. “Effect of Media Components and Culture Conditions on Ergosterol Content of *Aspergillus oryzae* .” Journal of Jiangxi Science & Technology Normal University 06: 87–92.

[fsn371347-bib-0016] Jin, J. S. , Z. T. Chen , and S. L. Li . 1999. “Expression of Macrophage Inflammatory Protein‐2 in Acute Lung Injury Induced by Endotoxin.” Chinese Journal of Pathophysiology 15, no. 11: 999–1002.

[fsn371347-bib-0017] Kanneganti, T. D. , M. Lamkanfi , Y. G. Kim , et al. 2007. “Pannexin‐1‐Mediated Recognition of Bacterial Molecules Activates the Cryopyrin Inflammasome Independent of Toll‐Like Receptor Signaling.” Immunity 26, no. 4: 433–443. 17433728 10.1016/j.immuni.2007.03.008

[fsn371347-bib-0018] Kim, E. K. , and E. J. Choi . 2010. “Pathological Roles of MAPK Signaling Pathways in Human Diseases.” Biochimica et Biophysica Acta‐General Subjects 4, no. 1802: 396–405. 10.1016/j.bbadis.2009.12.009 20079433

[fsn371347-bib-0019] Kyriakis, J. M. , and J. Avrush . 2001. “Mammalian Mitogen‐Activated Protein Kinase Signal Transduction Pathways Activated by Stress and Inflammation.” Physiological Reviews 2, no. 81: 807–869. 10.1152/physrev.2001.81.2.807 11274345

[fsn371347-bib-0021] Liu, C. , Z. W. Gao , H. P. Wang , et al. 2020. “Study on the Role of Artemisinin in Regulating the Proliferation and Migration of Carcharide‐Carcharide‐Cell.” Progress in Modern Biomedicine 20, no. 9: 6.

[fsn371347-bib-0022] Lou, L. , C. B. Chen , Y. Zhang , Q. L. Wan , H. Wang , and S. M. Wang . 2020. “Analysis of the Contents of Ergosterol and Ergosterone in Different Fermentation Products of *Marasmius epiphyllus* .” Special Wild Economic Animal and Plant Research 42, no. 4: 55–59.

[fsn371347-bib-0023] McGettrick, A. F. , and L. A. J. O'Neill . 2004. “The Expanding Family of MyD88‐Like Adaptors in Toll‐Like Receptor Signal Transduction.” Molecular Immunology 41, no. 6–7: 577–582. 15219996 10.1016/j.molimm.2004.04.006

[fsn371347-bib-0024] Miyake, K. 2004. “Endotoxin Recognition Molecules, Toll‐Like Receptor 4‐MD‐2.” Seminars in Immunology 16, no. 1: 11–16. 14751758 10.1016/j.smim.2003.10.007

[fsn371347-bib-0025] Nowak, R. , N. Nowacka‐Jechalke , W. Pietrzak , and U. Gawlik‐Dziki . 2022. “A New Look at Edible and Medicinal Mushrooms as a Source of Ergosterol and Ergosterol Peroxide—UHPLC‐MS/MS Analysis.” Food Chemistry 369, no. 1: 130927. 34461517 10.1016/j.foodchem.2021.130927

[fsn371347-bib-0026] O'Neill, L. A. J. , and A. G. Bowie . 2007. “The Family of Five: TIR‐Domain‐Containing Adaptors in Toll‐Like Receptor Signalling.” Nature Reviews Immunology 7, no. 5: 353–364. 10.1038/nri2079 17457343

[fsn371347-bib-0027] Qiao, M. F. , N. Y. Ji , X. H. Liu , F. Li , and Q. Z. Xue . 2010. “Asporyergosterol, A New Steroid From an Algicolous Isolate of *Aspergillus oryzae* .” Natural Product Communications 5, no. 10: 1575–1578. 21121251

[fsn371347-bib-0028] Qiao, M. F. , Y. W. Yi , and J. Deng . 2017. “Steroids From an Endophytic *Eurotium rubrum* Strain.” Chemistry of Natural Compounds 53: 678–681.

[fsn371347-bib-0029] Song, M. J. , H. Y. Bao , L. Tu , and Y. Li . 2015. “Analysis of the Antitumor Activity and Structure Activity Relationship of Four Steroids in *Sphingomonas elliptica* .” Journal of Fungi 34, no. 2: 293–300.

[fsn371347-bib-0030] Udenigwe, C. C. , J. Y. Je , Y. S. Cho , and R. Y. Yada . 2013. “Almond Protein Hydrolysate Fraction Modulates the Expression of Proinflammatory Cytokines and Enzymes in Activated Macrophages.” Food & Function 4, no. 5: 777–783. 23575976 10.1039/c3fo30327f

[fsn371347-bib-0031] Xu, W. Y. , A. Li , S. S. Mu , D. Hu , and S. M. Wang . 2019. “Simultaneous Determination of Ergosterol and Ergosterol Contents in 19 Medicinal Abdominal Bacteria Using HPLC Dual Wavelength Method.” Lishizhen Medicine and Materia Medica Research 30, no. 4: 1002–1005.

[fsn371347-bib-0032] Yang, D. J. , Y. Y. Chang , H. W. Lin , Y. C. Chen , S. H. Hsu , and J. T. Lin . 2014. “Inhibitory Effect of Litchi ( *Litchi chinensis* Sonn.) Flower on Lipopolysaccharide‐Induced Expression of Proinflammatory Mediators in RAW264.7 Cells Through NF‐κB, ERK, and JAK2/STAT3 Inactivation.” Journal of Agricultural and Food Chemistry 62, no. 15: 3458–3465. 24641487 10.1021/jf5003705

[fsn371347-bib-0033] Yuan, H. W. , J. Zhang , X. T. Zhang , et al. 2020. “Optimization of Fermentation Conditions of Brewed Rice Wine.” China Brewing 39, no. 12: 36–41.

[fsn371347-bib-0034] Zhang, L. , X. W. Zhu , K. He , J. P. Gong , and Z. L. Peng . 2021. “Investigation of the Mechanism of NLRP 3 Inflammasome Regulation Mediated by Autophagy.” Journal of Chongqing Medical University 50, no. 4: 541–546.

[fsn371347-bib-0035] Zhao, M. 2013. “TLR4‐Mediated Activation and Uptake of Oxidized Low Density Lipoprotein by Human Mononuclear Macrophages and Inhibitory Effect of Recombinant Oxidized Low Density Lipoprotein Monoclonal Antibodies.” Chinese Journal of Arteriosclerosis 21, no. 9: 1.

[fsn371347-bib-0036] Zhao, R. H. , X. L. He , and Q. Tian . 2020. “Research Advancement on Liquid Fermentation and the Application of Morchella Mycelia.” Food Research and Development 41, no. 12: 190–195.

[fsn371347-bib-0037] Zhao, Y. Y. , R. C. Lin , W. J. Sun , and R. M. Xie . 2011. “The Application of Ergostaerone in the Preparation of Drugs for Treating Chronic Renal Failure.” CN201010137836.8.

[fsn371347-bib-0038] Zhong, H. H. , H. SuYang , H. Erdjument‐Bromage , P. Tempst , and S. Ghosh . 1997. “The Transcriptional Activity of NF‐κB Is Regulated by the IκB‐Associated PKAc Subunit Through a Cyclic AMP–Independent Mechanism.” Cell 89, no. 3: 413–424. 9150141 10.1016/s0092-8674(00)80222-6

[fsn371347-bib-0039] Zhou, H. Y. , E. M. Shin , L. Y. Guo , et al. 2008. “Anti‐Inflammatory Activity of 4‐Methoxyhonokiol Is a Function of the Inhibition of iNOS and COX‐2 Expression in RAW 264.7 Macrophages via NF‐κB, JNK and p38 MAPK Inactivation.” European Journal of Pharmacology 586, no. 1–3: 340–349. 18378223 10.1016/j.ejphar.2008.02.044

