# Supplementary material for: Fermentation Optimization of Ergosta‐4,6,8(14),22‐Tetraen‐3‐One From Aspergillus oryzae and Its Anti‐Inflammatory Mechanism via Multi‐Pathway Inhibition of MyD88/NF‐κB/MAPK/NLRP3 Signaling
Source: Food Sci Nutr. 2025 Dec 15;13(12):e71347. doi: 10.1002/fsn3.71347 (PMC12703800; doi:10.1002/fsn3.71347)
Supplement: Supplementary file 1 — Figure S1: Effect of different carbon‐to‐nitrogen ratios in culture medium on ETO production by A.oryzae. Figure S2: Fungal culture on PDA medium. Figure S3: ETO standard curve. Figure S4: The stimulating effects of different concentrations of LPS. [file FSN3-13-e71347-s001.docx]

**SUPPLEMENTARY MATERIAL**

**Fermentation Optimization of Ergosta-4,6,8(14),22-tetraen-3-one from Aspergillus oryzae and Its Anti-Inflammatory Mechanism via Multi-Pathway Inhibition of MyD88/NF-κB/MAPK/NLRP3 Signaling**

**Bingye Yang^1,2,*^∣ Shining Liu^1,2^∣ Yu-Wei Chang^3^∣ Chao Yi^1,2^∣ Minxin You^1,2^∣ Yung-Husan Chen^1,2,*^**

^1^ Xiamen Key Laboratory of Natural Products Resources of Marine Medicine, Xiamen Medical College, Xiamen 361023, China; email: [liushining@xmmc.edu.cn](mailto:202005480025@xmmc.edu.cn) (S.L.); [201400080002@xmmc.edu.cn](mailto:201400080002@xmmc.edu.cn) (C.Y.); [202205480025@xmmc.edu.cn](mailto:202205480025@xmmc.edu.cn) (M.Y.); llz@xmmc.edu.cn(L.L.)

^2^ Fujian Provincial University Marine Biomedical Resources Engineering Research Center, Xiamen Medical College, Xiamen 361023, China

^3^ Department of Food Science, National Taiwan Ocean University, Keelung 20224, Taiwan; email: [bweichang@mail.ntou.edu.tw](mailto:bweichang@mail.ntou.edu.tw) (Y.-W.C)

* Correspondence: [cyxuan@xmmc.edu.cn](mailto:cyxuan@xmmc.edu.cn) (Y.-H.C.); [yby@xmmc.edu.cn](mailto:yby@xmmc.edu.cn) (B.Y.)

**Contents**

**Figure S1.** Effect of different carbon-to-nitrogen ratios in culture medium on ETO production by A.oryzae.

**Figure S2.** Fungal culture on PDA medium.

**Figure S3.** ETO standard curve.

**Figure S4.** The stimulating effects of different concentrations of LPS.


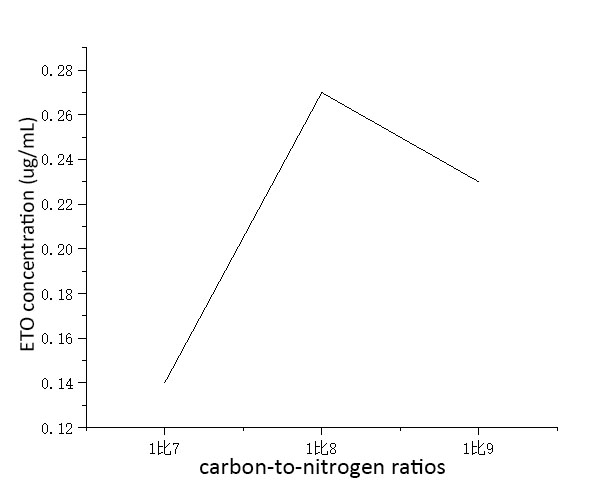


**Figure S1.** Effect of different carbon-to-nitrogen ratios in culture medium on ETO production by A.oryzae.

**
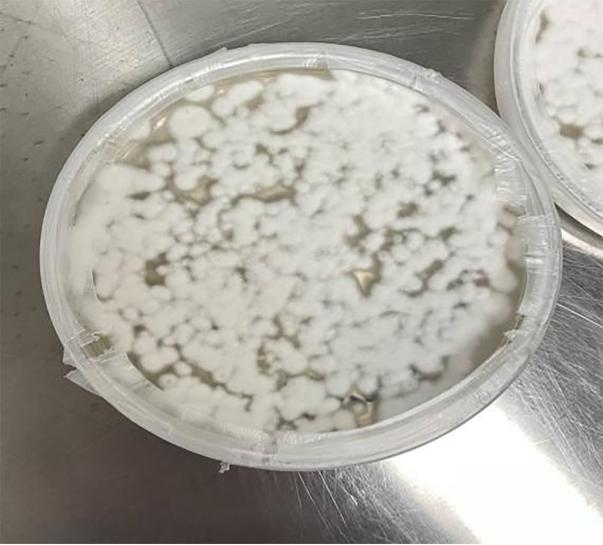

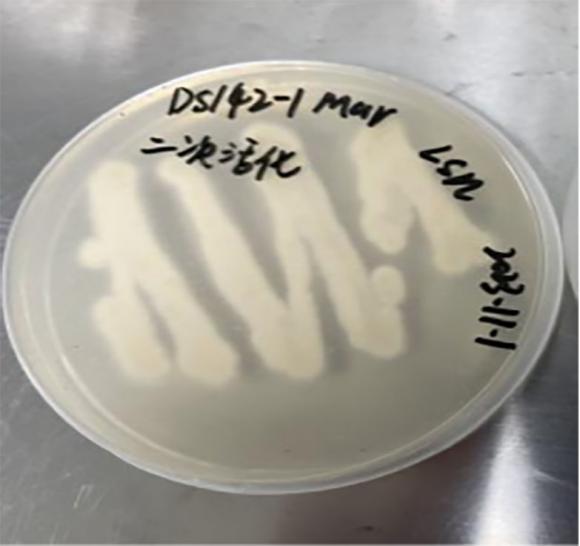
**

**Figure S2**. Fungal culture on PDA medium.


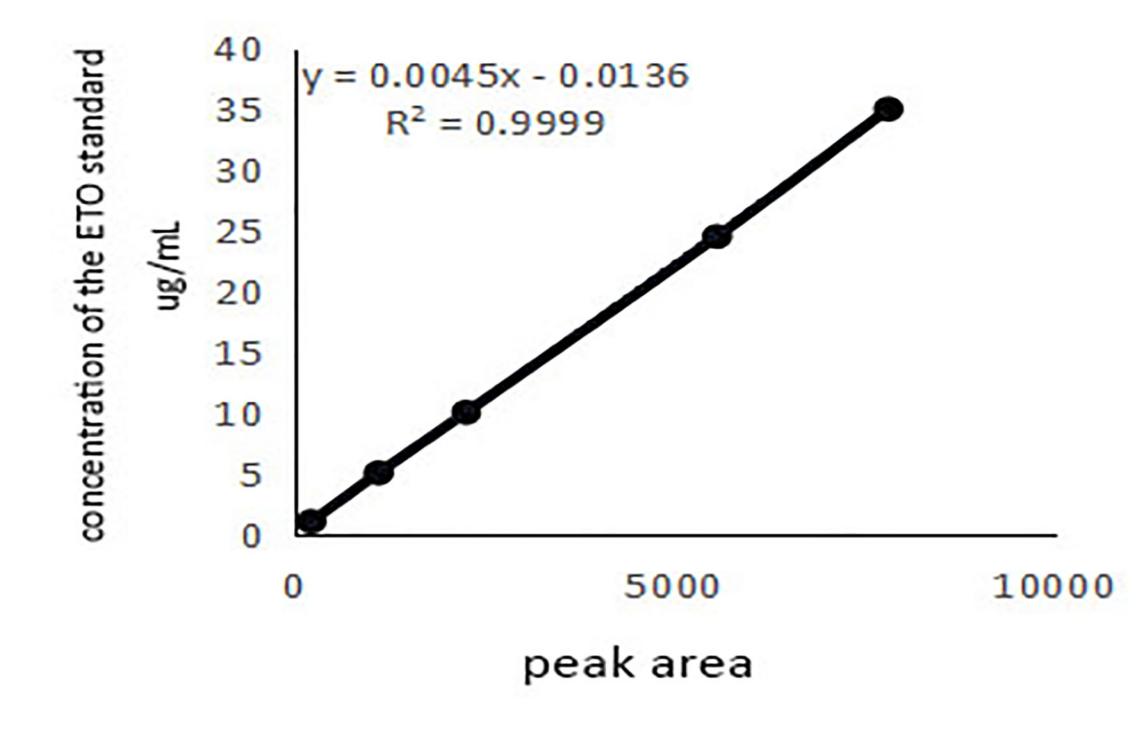


**Figure S3**. Standard curve of ETO


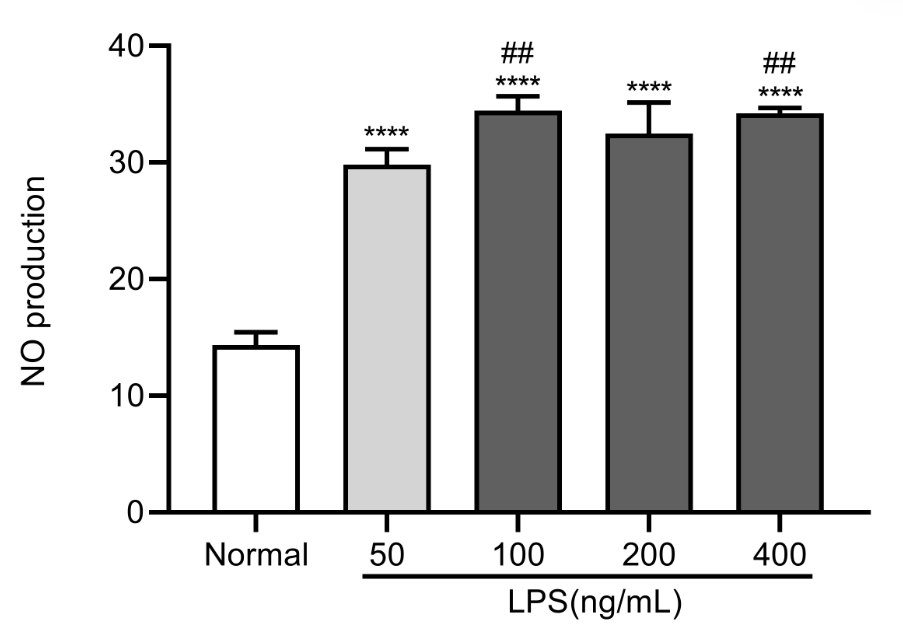


**Figure S4.** The stimulating effects of different concentrations of LPS.
